# Edoxaban‐induced enterocolitis: The first case report demonstrating distinct endoscopic and histological features

**DOI:** 10.1002/deo2.70142

**Published:** 2025-08-01

**Authors:** Katsuya Endo, Jun Yamada, Tomofumi Katayama, Yuki Yoshino, Daisuke Fukushi, Akinobu Koiwai, Takayuki Kogure, Morihisa Hirota, Kennichi Satoh

**Affiliations:** ^1^ Department of Gastroenterology Tohoku Medical and Pharmaceutical University Miyagi Japan

**Keywords:** colonoscopy, direct oral anticoagulants, edoxaban, enterocolitis, gastrointestinal hemorrhage

## Abstract

Direct oral anticoagulants (DOACs), including edoxaban, are widely used for stroke prevention in atrial fibrillation and venous thromboembolism. While gastrointestinal bleeding and diarrhea are recognized adverse effects, DOAC‐induced enterocolitis has not been established as a distinct clinical entity. We report the first case of edoxaban‐induced enterocolitis in a 75‐year‐old woman who developed bloody diarrhea and anorexia five days after starting edoxaban. Ileocolonoscopy revealed scattered redness, ulcers, and erosions in the terminal ileum, with diffuse edema and submucosal bleeding from the transverse colon to the rectum. Histopathology showed villous atrophy, lymphatic dilation, and lymphocyte‐predominant infiltration in the ileum, along with crypt atrophy, mucosal edema, and hemorrhages in the colon. These findings were inconsistent with infectious, ischemic, vasculitic, or inflammatory bowel diseases, suggesting a drug‐induced etiology. Given the acute onset and unique endoscopic and histopathological findings, edoxaban‐induced enterocolitis was suspected. The patient's symptoms resolved three days after discontinuing edoxaban, and a follow‐up ileocolonoscopy after 3 months showed complete mucosal healing. In accordance with the clinical course, we ultimately diagnosed this case as edoxaban‐induced enteritis. Given the widespread use of DOACs, similar cases may be underrecognized, as unexplained bloody diarrhea in these patients often lacks detailed endoscopic evaluation. Further case reports and studies are needed to establish DOAC‐induced enteritis as a distinct clinical entity. This case serves as a critical first step in recognizing DOAC‐induced enterocolitis and highlights the need for increased awareness among clinicians.

## INTRODUCTION

Direct oral anticoagulants (DOACs), including edoxaban, are widely used for stroke prevention in atrial fibrillation and for venous thromboembolism. While gastrointestinal (GI) bleeding and diarrhea are well‐documented complications of DOAC therapy, their full spectrum of GI toxicity remains unclear.^1^ Among anticoagulant‐associated GI complications, DOAC‐induced enterocolitis has not been previously described as a distinct clinical entity. Although sporadic reports have linked DOACs to non‐specific diarrhea, cases with well‐characterized endoscopic and histopathological features have yet to be documented.

Here, we report the first case of edoxaban‐induced enterocolitis, characterized by unique endoscopic and pathological findings, which were resolved completely after discontinuing edoxaban. This case highlights the need for greater awareness of potential enteric complications associated with DOACs.

## CASE REPORT

**FIGURE 1:**
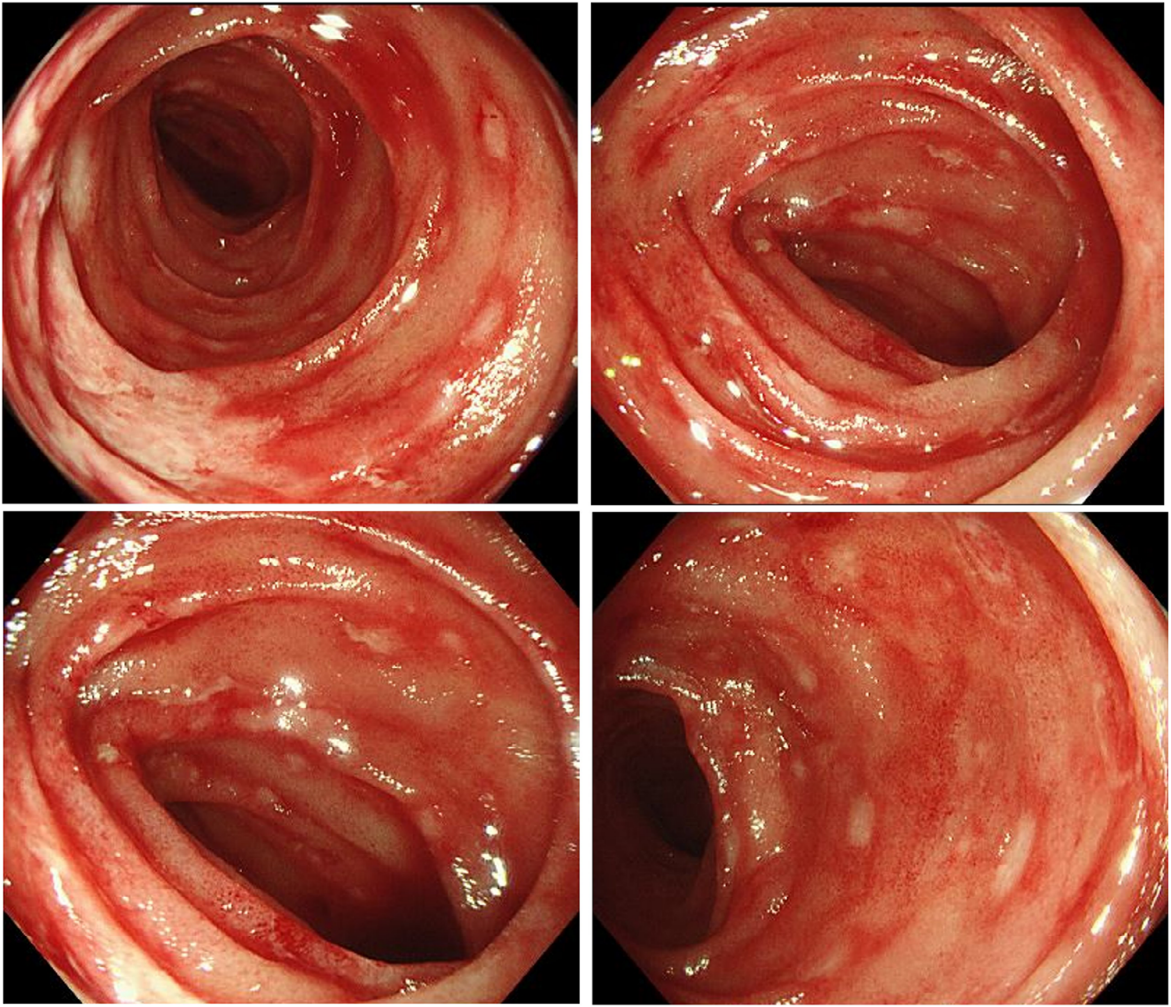
Endoscopic findings of the terminal ileum on the 11th day of edoxaban treatment. Scattered redness, shallow ulcers with irregular shape, and small erosions with spontaneous bleeding were observed.

**FIGURE 2:**
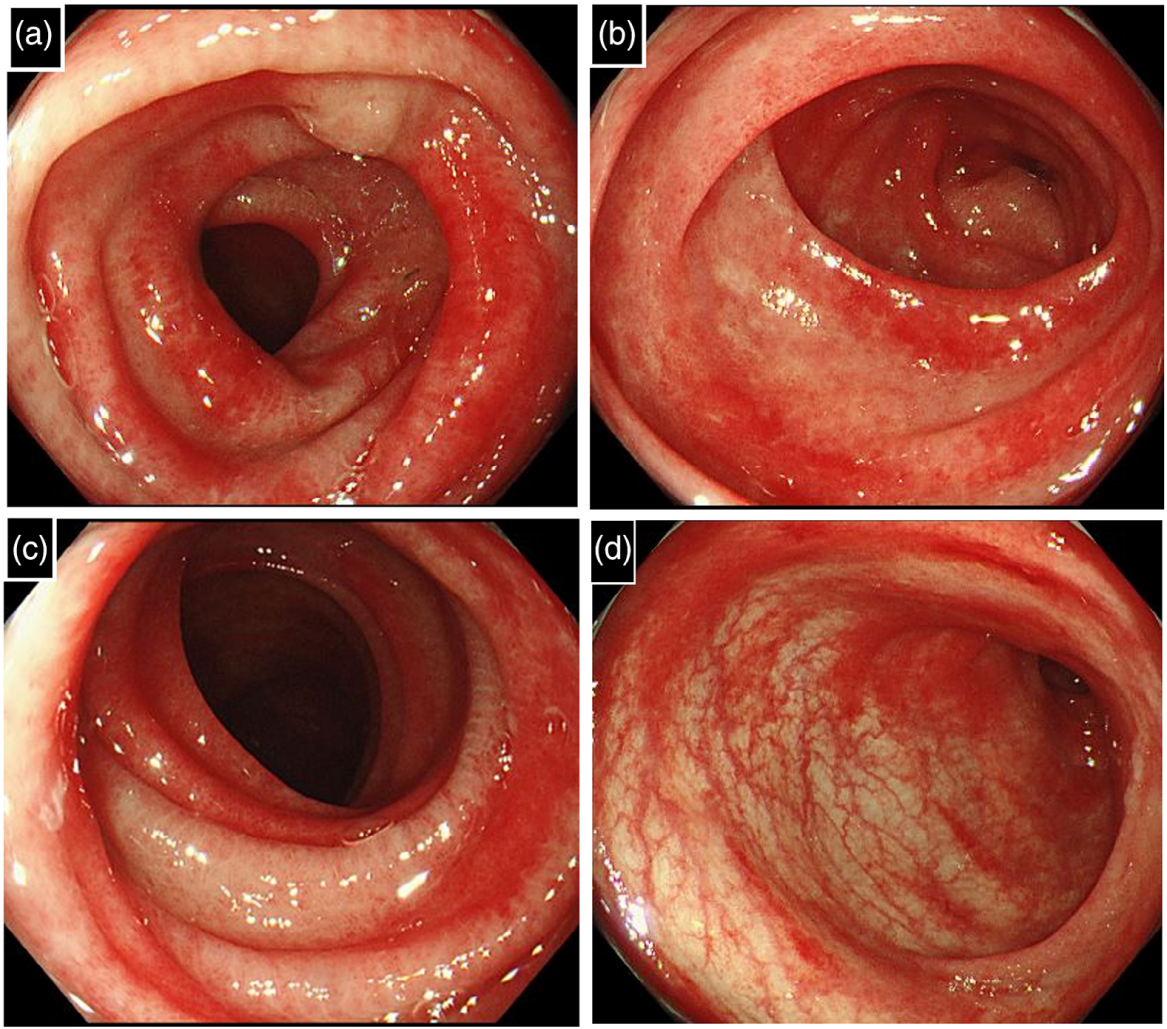
Endoscopic findings of the colon on the 11th day of edoxaban treatment. (a) Transverse colon, (b) splenic flexure, (c) descending colon, and (d) sigmoid colon. Diffuse edema and intense redness which suggests submucosal bleeding were observed.

**FIGURE 3:**
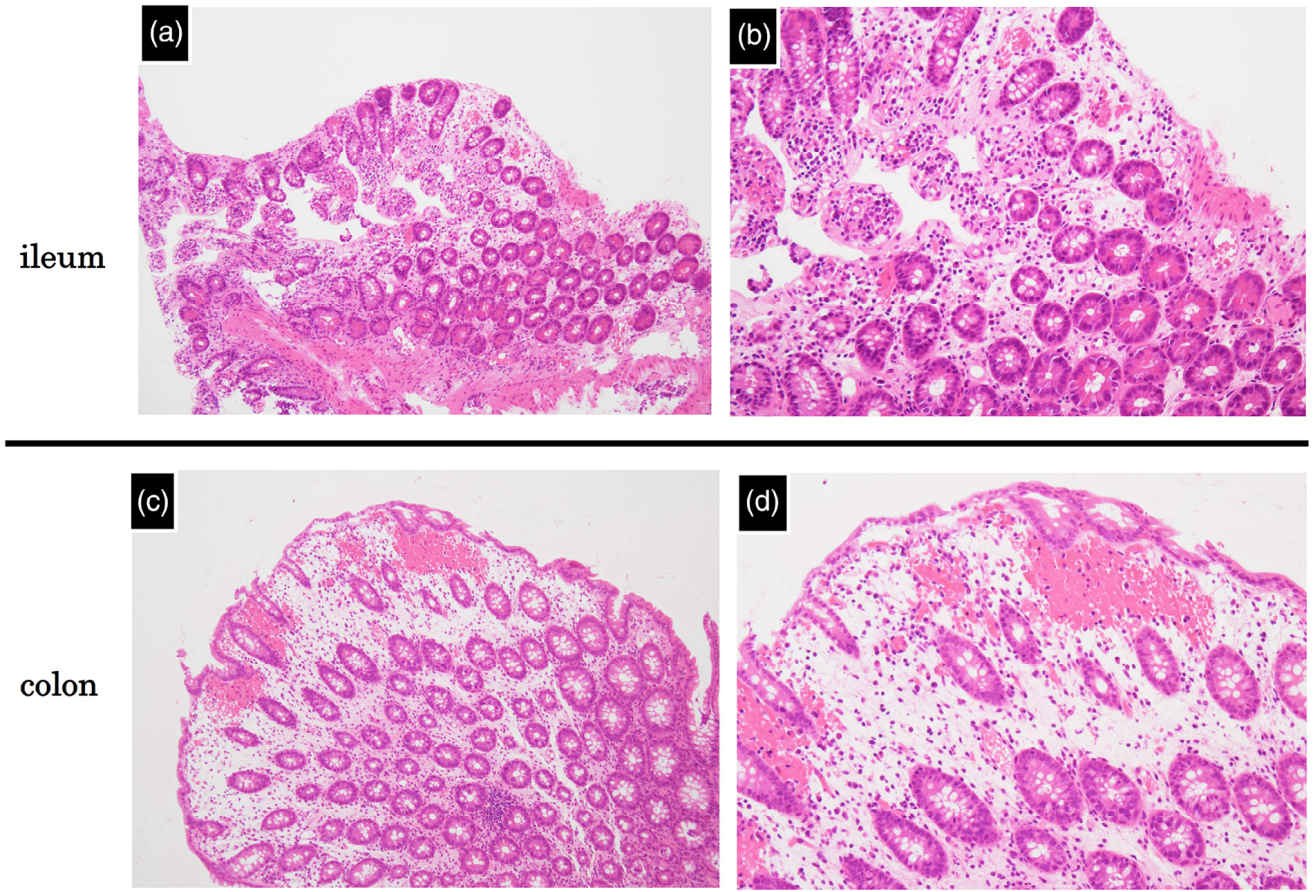
Histopathological findings of the ileum and descending colon. (a, b) In the terminal ileum, villous atrophy, lymphatic vessel dilation, and lymphocyte‐predominant infiltration were observed. (c, d) In the descending colon, crypt atrophy, lymphocyte‐predominant inflammatory infiltration, and mucosal edema with hemorrhage were noted.

A 75‐year‐old woman was admitted to the cardiology department of our hospital for the treatment of atrial fibrillation with heart failure. Edoxaban was initiated to prevent thromboembolism three days after admission. On the fifth day of edoxaban administration, the patient developed bloody diarrhea and anorexia. She was referred to the gastroenterology department on the 10th day of treatment. At the time of consultation, she had four to five episodes of bloody diarrhea per day. Her vital signs were stable, except for an irregular pulse of 80 beats per minute. Abdominal examination revealed no significant abnormalities. Laboratory findings indicated marked hypoalbuminemia (3.2 g/dL), hypokalemia (3.1 mmol/L), and slightly elevated C‐reactive protein level (0.32 mg/dL). The stool culture showed only normal flora, and *Clostridioides difficile* toxin and glutamate dehydrogenase (GDH) antigen tests were negative. Contrast‐enhanced abdominal CT revealed thickening of the pelvic ileum and the colonic wall from the left transverse colon to the rectum, as shown in Figure S1. Ileocolonoscopy, performed on the 11th day of edoxaban treatment, showed scattered redness, shallow ulcers with an irregular shape, and small erosions with spontaneous bleeding in the terminal ileum (Figure 1). Additionally, diffuse edema and intense redness, suggestive of submucosal bleeding, were observed from the left transverse colon to the rectum (Figure 2). Histological examination of biopsy specimens revealed inflammation, distinct from infectious or ischemic enteritis. Pathological findings of the terminal ileum included villous atrophy, lymphatic vessel dilation, and lymphocyte‐predominant infiltration (Figure 3a,b). In the descending colon, crypt atrophy, lymphocyte‐dominant inflammatory infiltration, and mucosal edema with hemorrhages were observed (Figure 3c,d).

**FIGURE 4:**
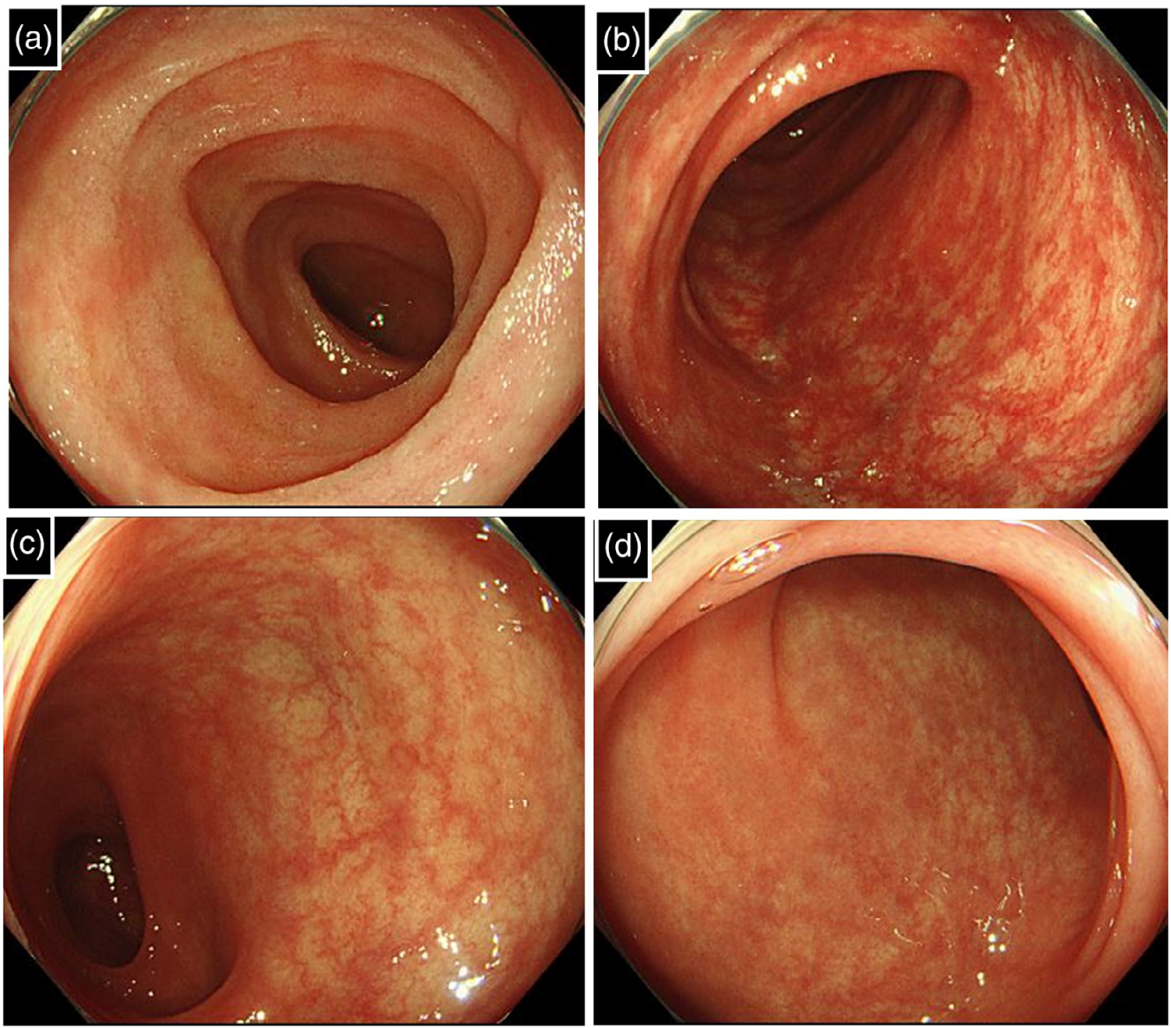
Ileocolonoscopic findings 3 months after discontinuing edoxaban. (a) Terminal ileum and (b–d) colon. Follow‐up ileocolonoscopy 3 months after discontinuing edoxaban demonstrated complete mucosal healing.

Given the acute symptom onset shortly after edoxaban initiation, along with the unique endoscopic and histological findings, drug‐induced enteritis due to edoxaban was suspected. Edoxaban was discontinued on day 11, and warfarin was substituted. The bloody stools resolved within 2–3 days, and the watery diarrhea gradually improved. Within 2 weeks of discontinuation, stool consistency normalized, and no recurrence of symptoms was observed. Follow‐up ileocolonoscopy 3 months after stopping edoxaban showed complete mucosal healing (Figure 4). Based on the clinical course, as well as the endoscopic and pathological findings, we ultimately diagnosed this case as edoxaban‐induced enteritis.

## DISCUSSION

DOACs, including edoxaban, are widely prescribed for the prevention of thromboembolic events in patients with atrial fibrillation and venous thromboembolism due to their favorable efficacy and safety profiles compared to warfarin. However, GI adverse effects, including bleeding, diarrhea, and dyspepsia, are well‐documented side effects of DOACs.^1^, ^2^, ^3^ Among these, GI bleeding is particularly concerning, with studies reporting an increased risk compared to warfarin, especially in the upper GI tract. Although diarrhea and other GI symptoms have been reported, DOAC‐induced enterocolitis remains an unrecognized entity, and no prior case reports have described enterocolitis with distinct endoscopic and histological findings following DOAC administration. In this report, we described the first case of edoxaban‐induced enterocolitis.

The patient developed bloody diarrhea shortly after edoxaban initiation, with characteristic and unique endoscopic and histopathologic findings. The endoscopic findings, characterized by scattered redness, shallow ulcers with an irregular shape, and small erosions in the terminal ileum, as well as diffuse edema and intense redness suggestive of submucosal hemorrhage in the colon, were distinct from those observed in other forms of enterocolitis.^4^ Histological examination revealed non‐specific inflammation with villous atrophy, lymphatic vessel dilation, and lymphocytic infiltration, findings that do not correspond to the characteristic patterns of other enterocolitis.^5^, ^6^ These endoscopic and pathological features do not align with known patterns of infectious, ischemic, inflammatory bowel disease‐related, or autoimmune colitis, further supporting the hypothesis of DOAC‐induced enteritis.

The diagnosis of drug‐induced enterocolitis is established by excluding other potential causes and confirming symptom resolution after drug discontinuation.^7^ Regarding the exclusion of other diseases, we carefully ruled out infectious, ischemic, inflammatory, and vasculitic etiologies through clinical, laboratory, imaging, and histological assessments. Infectious colitis, including *C. difficile* infection, was excluded based on negative stool cultures and toxin assays. Inflammatory bowel disease was considered unlikely given the acute onset and absence of chronic inflammatory changes in histology. Similarly, ischemic colitis, which typically presents segmental involvement and fibrinoid necrosis, was not suggested by the endoscopic and pathological findings. Furthermore, vasculitis‐related enterocolitis, such as that seen in polyarteritis nodosa or microscopic polyangiitis, was deemed unlikely given the absence of systemic vasculitic manifestations and the non‐vasculitic nature of the histological findings. Another important criterion for diagnosing drug‐induced enterocolitis is whether withdrawal of the offending drug alone leads to the resolution of symptoms. In this case, rapid symptomatic improvement and complete endoscopic healing were achieved following edoxaban discontinuation, without any additional intervention. This clinical course strongly supports a causal relationship and confirms the diagnosis of edoxaban‐induced enterocolitis.

The exact mechanism of edoxaban‐induced enterocolitis remains unclear. However, we speculate that its pathology cannot be explained solely by anticoagulation‐related bleeding. Instead, features such as lymphocyte‐predominant infiltration suggest additional mechanisms, possibly involving immune‐mediated mucosal injury. In this context, its histopathology also differs from other drug‐induced enteropathies, such as non‐steroidal anti‐inflammatory drug (NSAID)‐induced enteropathy, which typically shows chronic architectural changes including submucosal fibrosis, crypt distortion, and epithelial apoptosis. In contrast, our case revealed acute injury with lymphocyte‐predominant infiltration, without evidence of fibrosis or chronic architectural changes. Notably, epithelial apoptosis—a typical feature of NSAID‐related injury—was not observed. These findings suggest a distinct pathological process and support the classification of edoxaban‐induced enterocolitis as a separate histopathological entity. Further studies are warranted to clarify the underlying pathophysiology.

To the best of our knowledge, this is the first reported case of edoxaban‐induced enteritis. Given the widespread use of DOACs, similar cases may already exist but remain unrecognized in clinical practice. Patients presenting with unexplained bloody diarrhea while on DOAC therapy may not always undergo detailed endoscopic evaluations, leading to underdiagnosis of this condition. Further case reports and studies are needed to establish DOAC‐induced enteritis as a distinct clinical entity. This case serves as a critical first step in establishing the concept of DOAC‐induced enterocolitis and highlights the need for increased awareness among clinicians.

## CONFLICT OF INTEREST STATEMENT

None.

## ETHICS STATEMENT

This study was conducted in accordance with the ethical principles of the Declaration of Helsinki for medical research involving human participants. This study was approved by the Ethics Committee of the Tohoku Medical and Pharmaceutical University (2024‐4‐087). Informed consent was obtained from the patient to be included in this study.

## Supporting information




**FIGURE S1 Contrast‐enhanced abdominal CT**. Contrast‐enhanced abdominal CT revealed thickening of the pelvic ileum and the colonic wall from the left transverse colon to the rectum.
