# Supplementary figures and images for: Edoxaban‐induced enterocolitis: The first case report demonstrating distinct endoscopic and histological features
Source: DEN Open. 2025 Aug 1;6(1):e70142. doi: 10.1002/deo2.70142 (PMC12315852; doi:10.1002/deo2.70142)

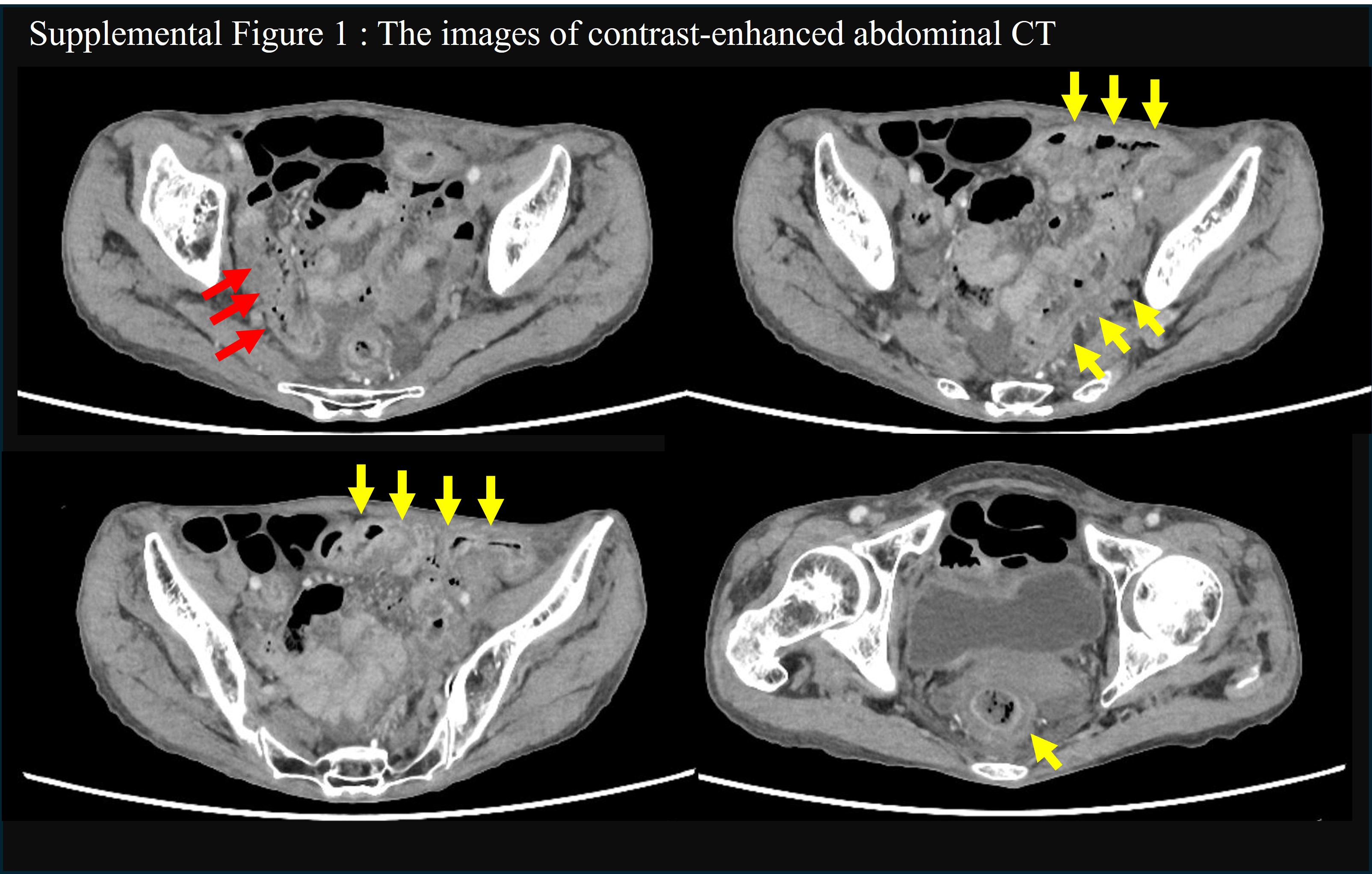

Supplement: Supplementary file 1 — FIGURE S1 Contrast‐enhanced abdominal CT. Contrast‐enhanced abdominal CT revealed thickening of the pelvic ileum and the colonic wall from the left transverse colon to the rectum. [file DEO2-6-e70142-s001.jpg]
